# Multiple Bone Metastases From Non‐Muscle Invasive Bladder Cancer Responding to Combination Therapy With Enfortumab Vedotin and Pembrolizumab: A Case Report

**DOI:** 10.1002/iju5.70119

**Published:** 2025-12-25

**Authors:** Shin Kanemoto, Shinkuro Yamamoto, Kosuke Hino, Kazuma Tsuboi, Mototsune Kakizaki, Yasuhiro Nishiyama, Satoshi Fukata, Ryoji Arata, Keiji Inoue, Noriaki Ono

**Affiliations:** ^1^ Department of Urology Kochi Health Science Center Kochi Japan; ^2^ Department of Urology Kochi Medical School Nankoku Japan; ^3^ Department of Pathology Kochi Health Science Center Kochi Japan

**Keywords:** carcinoma in situ, enfortumab vedotin and pembrolizumab, multiple bone metastases, non‐muscle invasive bladder cancer

## Abstract

**Introduction:**

We report a case in which combination therapy with enfortumab vedotin (EV) and pembrolizumab proved effective in treating non‐muscle‐invasive bladder cancer (NMIBC) accompanied by multiple bone metastases.

**Case Presentation:**

A 61‐year‐old man with NMIBC underwent eight transurethral resections of bladder tumor (TURBTs) at another hospital over 4 years. During the eighth TURBT, carcinoma in situ was detected, and the patient was treated with intravesical Bacillus Calmette‐Guérin (BCG) therapy. A computed tomography scan performed at our hospital 1.5 years after BCG therapy revealed multiple osteosclerotic lesions. Cystoscopy and bladder magnetic resonance imaging revealed no obvious tumorous lesions. An incisional biopsy of the sternum confirmed the diagnosis of multiple bone metastases originating from NMIBC. The patient was started on combination therapy with EV and pembrolizumab. After 5 months, bone scintigraphy revealed decreased accumulation.

**Conclusion:**

Combination therapy with EV and pembrolizumab was also effective in treating NMIBC with multiple bone metastases.

**Summary**

- Multiple bone metastases without local progression are extremely rare in bladder cancer.
- Careful follow‐up of high‐risk NMIBC should include the possibility of distant metastasis.
- Combination therapy with enfortumab vedotin and pembrolizumab was effective in treating NMIBC with multiple bone metastases.

## 1 Introduction

Non‐muscle invasive bladder cancer (NMIBC) accounts for approximately 70% of all bladder cancer cases and generally has a relatively favorable prognosis [1]. The standard treatment is transurethral resection of bladder tumor (TURBT), often followed by a second TUR or intravesical infusion therapy with anticancer drugs or Bacillus Calmette‐Guérin (BCG), depending on the risk of progression. Although NMIBC rarely metastasizes, vigilance is crucial due to the potential for distant metastasis. On September 24, 2024, enfortumab vedotin (EV) and pembrolizumab combination therapy was approved in Japan as a new treatment for unresectable urothelial carcinoma (UC). We report a case of multiple bone metastases from NMIBC, detected through multiple osteosclerotic lesions, successfully treated with EV and pembrolizumab.

## 2 Case Presentation

**FIGURE 1:**
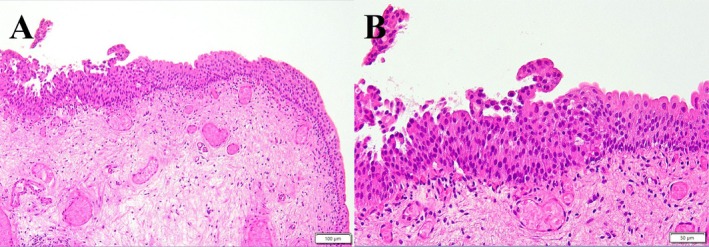
Pathological findings from the eighth transurethral resections of bladder tumor. (A) Hematoxylin and Eosin (H&E)‐stained section at low magnification (×10) showing the subepithelial connective tissue (lamina propria; the muscularis propria was not included) with no evidence of invasion. (B) H&E‐stained section at higher magnification (×20) showing focal proliferation of urothelial cells with mild nuclear enlargement, hyperchromasia, irregular shape, and loss of polarity. The degree of atypia is mild, and no definite infiltration is observed in the specimen.

**FIGURE 2:**
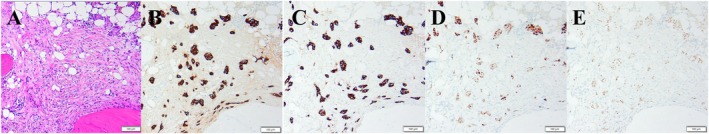
(A) H & E‐stained section showing atypical cells with pale eosinophilic cytoplasm arranged in alveolar and fascicular arrangements (magnification 10×). IHC‐stained sections showing positivity for (B) CK‐AE/AE3; (C) CK7; (D) CK20; (E) GATA3. CK‐AE/AE3, CK7, and CK20 are epithelial cell markers, while GATA3 indicates differentiation into urothelium. The findings are suggestive of urothelial carcinoma.

**FIGURE 3:**
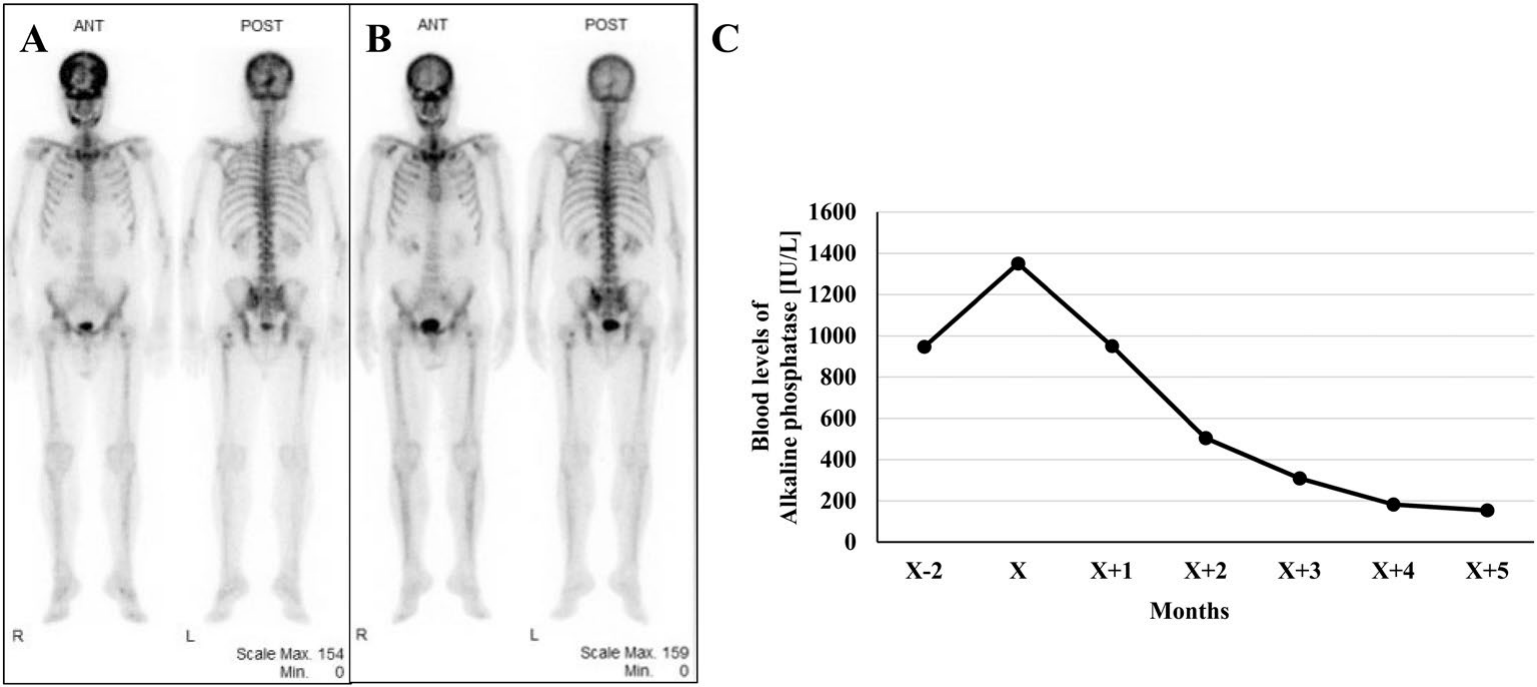
Bone scintigraphy. (A) Bone scintigraphy before drug treatment shows increased accumulation in bones throughout the body, from the skull to both femurs. BSI: 5.27%. (B) Bone scintigraphy after drug treatment shows significantly reduced accumulation in bone metastatic lesions. BSI: 2.12%. (C) Changes in blood levels of alkaline phosphatase before and after drug treatment. X indicates the month treatment was started. ALP was elevated before the initiation of enfortumab vedotin and pembrolizumab combination therapy, but gradually decreased after treatment initiation.

A 61‐year‐old man with superficial bladder cancer underwent TURBT at a previous hospital. Low‐grade UC was identified. Although some interstitial invasion was noted, there was no evidence of lymphovascular invasion (LVI) or muscularis propria involvement. No metastasis was present, and the patient was diagnosed with pT1N0M0 NMIBC. He had been receiving cyclosporine to treat psoriasis vulgaris; therefore, BCG therapy was contraindicated. Given the low‐grade nature of the tumor, he was managed with close monitoring. Thereafter, the tumor recurred repeatedly in the bladder, necessitating seven TURBT procedures over a 3‐year period. The third TURBT revealed only atypical cells without definitive UC; however, the second, fourth, fifth, and seventh TURBTs demonstrated high‐grade UC, all staged pTa. The sixth TURBT showed low‐grade UC with slight interstitial invasion (pT1). However, no LVI or muscle invasion was identified in any of the TURBT specimens. Nine months after the seventh TURBT, urine cytology became positive. Cystoscopy did not reveal any papillary lesions; however, diffuse mucosal erythema was observed. The eighth TURBT was subsequently performed for diagnostic biopsy, which revealed carcinoma in situ (CIS) (Figure 1). Even if considered CIS, atypia was minimal and could alternatively be interpreted as dysplasia. By this time, the patient had discontinued immunosuppressive therapy. He then received six rounds of intravesical BCG therapy. Follow‐up urine cytology and cystoscopy showed no signs of recurrence or upper urinary tract malignancy. Computed tomography (CT) performed 3 months after BCG therapy showed no radiological evidence of disease recurrence. However, a CT scan performed at our hospital 1.5 years after BCG therapy revealed multiple osteosclerotic lesions. Concurrently, blood tests revealed elevated alkaline phosphatase (ALP) level (947 IU/L). Bone scintigraphy showed a “super bone scan” pattern. 18F‐fluorodeoxyglucose positron emission tomography/CT showed no other lesions. A CT‐guided needle biopsy of the iliac bone was performed, and based on findings, bone metastasis of UC was suspected. Cystoscopy showed no tumorous lesions, and urine cytology was class II. Bladder magnetic resonance imaging also showed no evidence of bladder cancer recurrence or local progression. Therefore, an incisional biopsy was performed from the sternum for further evaluation. Histopathological examination revealed proliferation of atypical cells with pale eosinophilic cytoplasm. Immunohistochemistry was positive for CK‐AE1/AE3, CK7, CK20, and GATA3, with weak positivity for uroplakin III (Figure 2). p63 and p40 showed focal positivity. The pathological results were consistent with bone metastasis of UC. Given the patient's history of NMIBC and the absence of other potential primary lesions, the patient was diagnosed with multiple bone metastases from bladder cancer. He was started on combination therapy with EV and pembrolizumab. After 5 months, bone scintigraphy showed an overall decrease in accumulation. The bone scan index decreased from 5.27% to 2.12%. ALP levels also showed a downward trend after the start of treatment, dropping to 158 IU/L within 4 months (Figure 3). No adverse events were observed, and treatment is currently ongoing.

## 3 Discussion

NMIBC accounts for approximately 70% of untreated bladder cancer cases [1]. TURBT is the standard treatment, and intravesical infusion of anticancer agents or BCG may be used to reduce the risk of recurrence or progression [2]. Despite high recurrence rates (40%–80%), NMIBC has a good prognosis. However, approximately 15% of patients develop local extension or metastasis [3]. Risk factors for progression to muscle‐invasive bladder cancer (MIBC) include T stage, pathologic grade, and the presence of CIS. Intravesical BCG therapy helps prevent or delay the progression of NMIBC, yet 6.2% develop MIBC with distant metastasis, and 2.9% develop distant metastasis without local progression [4]. The hypothesized mechanisms of distant metastasis without local invasion include lymphatic drainage from the bladder neck tumor, tumor cell contamination due to bladder perforation during TURBT, and intravascular seeding during TURBT [5]. Identification of LVI in TURBT specimens carries a risk of pathological upstaging [6]. Distant metastases from NMIBC most commonly occur in the lungs, bones, and lymph nodes [7]. However, only bone metastases were observed in the present case. It has been reported that direct metastasis to bone can occur through the Batson venous plexus, a valveless venous network in the pelvis and spine that allows tumor cells to bypass the liver and lungs [8]. Although LVI was not confirmed in any of the eight TURBT specimens, its presence cannot be completely ruled out. Alternatively, repeated TURBT procedures may have facilitated intravascular seeding, leading to hematogenous dissemination and subsequent bone metastasis via the Batson venous plexus.

Metastases are detected an average of 3.5 years after the first TURBT, highlighting the need for long‐term extrapelvic evaluation, especially in high‐risk NMIBC patients, even years after initial treatment [7]. According to the NCCN guidelines, annual upper urinary tract surveillance is recommended for patients with high‐risk NMIBC, whereas routine bone evaluation and chest CT are not included in standard follow‐up protocols [9]. Regular imaging evaluations were not performed in the present case, and the condition became apparent only after multiple bone metastases had developed. Based on this experience, we suggest that in patients with a history of T1 disease, CIS, or recurrent TURBTs, annual CT scans for at least 5 years should be considered to facilitate early detection of distant metastases.

The EV‐302 trial demonstrated the clinical efficacy of EV and pembrolizumab combination therapy [10]. According to the NCCN guidelines, the EV plus P combination is currently listed as the preferred regimen for primary systemic therapy in patients with locally advanced or metastatic bladder cancer. Although GC plus nivolumab and GC followed by avelumab maintenance are also available, they are categorized as “other recommended regimens.” [9] EV is an antibody‐drug conjugate targeting nectin‐4, while pembrolizumab blocks PD‐1, preventing PD‐1/PD‐L1‐mediated T cell inactivation and promoting antitumor immune responses [11]. Their complementary mechanisms support the rationale for combining them in cancer treatment. Nectin‐4 is expressed in approximately 87% of NMIBC cases [12], with particularly high expression rates observed in CIS and high‐grade UC [13]. Nectin‐4 positivity in UC of the bladder varies by stage, with higher rates in metastatic and non‐muscle‐invasive cases compared to muscle‐invasive cases [14].

For EV alone, a relationship between nectin‐4 expression and therapeutic effect has been suggested, indicating potential effectiveness in treating metastatic NMIBC [15]. Several reports have described the efficacy of pembrolizumab monotherapy for NMIBC [16, 17]. Taking these reports into consideration and the patient's relatively younger age within this demographic, we used a combination of EV and pembrolizumab in this patient. Given the reported efficacy of pembrolizumab monotherapy and EV monotherapy in NMIBC, the combination of EV and pembrolizumab may offer even greater therapeutic potential, as observed in this case. Although it is unclear whether the distant metastasis in our patient was related to CIS, this case also demonstrates the usefulness of EV and pembrolizumab combination therapy for NMIBC.

## 4 Conclusion

Combination therapy with EV and pembrolizumab was effective in NMIBC with multiple bone metastases.

## Ethics Statement

The authors have nothing to report.

## Consent

Written informed consent was obtained from the patient.

## Conflicts of Interest

K.I. is a member of the Editorial Board of the *International Journal of Urology* and a coauthor of this article. To avoid any potential bias, K.I. was excluded from all editorial decision‐making regarding the acceptance of this article.

## Data Availability

The authors have nothing to report.
